# High survivorship and excellent functional outcome in third‐generation patellofemoral arthroplasty

**DOI:** 10.1002/jeo2.70287

**Published:** 2025-06-15

**Authors:** Mustafa Hariri, Hannah Schwab, Kevin‐Arno Koch, Paul Mick, Timo Nees, Johannes Weishorn, Tilman Walker, Tobias Reiner

**Affiliations:** ^1^ Department of Orthopaedics Heidelberg University Hospital Heidelberg Germany

**Keywords:** patellofemoral arthroplasty (PFA), knee arthroplasty, knee replacement

## Abstract

**Purpose:**

This study presents the short‐ to mid‐term implant survival rates, along with the clinical and radiographic outcomes, in a consecutive series of patients who underwent third‐generation patellofemoral arthroplasty (PFA) at a non‐designer centre. Additionally, it explores the impact of prior surgery on clinical outcomes following PFA.

**Methods:**

A retrospective analysis of prospectively collected data was conducted for 27 PFAs performed in 23 patients between 2016 and 2023 using the Gender Solutions® Patello‐Femoral Joint System. Patients had a mean follow‐up of 4.3 years, with clinical assessments including the Oxford Knee Score (OKS), Visual Analogue Scale (VAS), Forgotten Joint Score (FJS), and activity levels (TAS, UCLA). Implant survivorship was analysed using Kaplan‐Meier estimators, with endpoints of revision and reoperation

**Results:**

The mean age at time of surgery was 51.7 ± 10.5 years, with 92.6% of patients being women. The mean body mass index (BMI) was 28.3 ± 4.5. The 5‐year implant survival rate was 100% for revisions and 96.3% for reoperations. Statistically significant improvements were observed in OKS (24.7 ± 8.0 to 39.2 ± 8.3, *p* < 0.001), VAS (7.1 ± 2.3 to 2.3 ± 2.8, *p* < 0.001) and range of motion (ROM) (122.1° ± 17° to 134.7° ± 6.8°, *p* = 0.007). Over 85% of patients achieved good to excellent OKS scores, with 92.3% reporting satisfaction. Patients with prior surgery on the affected knee showed higher satisfaction and greater ROM improvement. Obesity was associated with minor reductions in ROM but did not significantly impact overall outcomes.

**Conclusion:**

The short‐ to mid‐term results following third‐generation PFA demonstrated high survivorship and excellent clinical outcomes in an independent series. Prior surgery and obesity were not associated with poorer clinical outcomes, supporting the consideration of PFA for these patients when appropriately indicated.

**Level of Evidence:**

Level III, retrospective cohort study.

**Abbreviations**
ACL: anterior cruciate ligament
AP: anteroposterior
BMI: body mass index
FU: follow‐up
MIS: minimally invasiv technique
MPFL: medial patellofemoral ligament
NJR: national joint registry
OA: osteoarthritis
OKS: oxford knee score
PFA: patellofemoral arthroplasty
PFOA: patellofemoral osteoarthritis
ROM: range‐of‐motion
SD: standard deviation
TAS: tegner activity score
TKA: total knee arthroplasty
TTO: tibial tubercle osteotomy
UCLA: The University of California Los Angeles activity scale
UKA: unicompartmental knee arthroplasty
VAS: visual analogue scale

## INTRODUCTION

Isolated patellofemoral osteoarthritis (PFOA) primarily affects the patellofemoral compartment of the knee, leading to joint degeneration and pain, particularly during activities such as stair climbing or rising from a seated position. The prevalence of isolated PFOA is substantial, impacting approximately 18% of the population, with a higher incidence observed in females [30].

In addition to patellar trauma from fractures, isolated PFOA is predominantly caused by patellofemoral instability, which can lead to full‐thickness loss of the articular cartilage in both the patella and trochlear groove [4, 15].

When joint‐preserving surgical approaches prove ineffective, treatment options for severe PFOA include total knee arthroplasty (TKA) or patellofemoral arthroplasty (PFA). PFA presents the advantage of preserving bone and ligaments, frequently resulting in superior functional outcomes compared to TKA [5, 20]. Furthermore, PFA facilitates easier conversion to TKA if necessary, which is advantageous for younger patients with a higher likelihood of requiring revision surgery during their lifetime [8].

Early designs of PFA had high failure and reoperation rates, primarily because these inlay prostheses were designed to replace only the worn cartilage of the trochlea and, therefore, failed to address underlying issues such as maltracking and trochlear dysplasia, which are commonly observed in cases of isolated PFOA [31, 32]. Onlay prostheses, on the other hand, are based on the principles of TKA and require complete resection of the trochlea [10]. As a result, they are not influenced by the native trochlear morphology [5]. Advancements in second‐generation PFA with onlay designs include a wider trochlear groove, an extended anterior flange, and improved instrumentation, all of which have contributed to promising early outcomes and reduced failure rates [36]. Despite these improvements, registry studies indicate that PFA failure rates remain higher than those of TKA, ranging from 7.6% to 30.3% at 5 years, depending on the specific prosthesis used [28]. The most common failure modes of PFA include the progression of tibiofemoral OA (42%), persistent pain (16%) and aseptic loosening (13%) [6].

To further improve outcomes in PFA, recent research has focused on the anatomical and biomechanical differences between genders, as approximately two‐thirds of isolated PFOA cases occur in females [34, 37]. This focus has led to the development of a third‐generation PFA onlay prosthesis with gender‐specific design features, including an asymmetric left‐right onlay and a wider trochlear groove angle to accommodate the anatomical variations between male and female knees. Additionally, the anterior flange is designed to be thinner and extends proximally, reducing overhang and enhancing patellofemoral contact, particularly in cases involving patella alta [27].

In addition to technical advancements, adherence to strict inclusion and exclusion criteria in patient selection has also contributed to improved outcomes [16]. Several studies have highlighted the importance of considering body mass index (BMI) and mental health status when evaluating candidates for PFA [12, 18]. However, the impact of prior surgery as a predictor of PFA outcomes remains uncertain.

This study examines the short‐ to mid‐term implant survival rates, as well as the clinical and radiographic outcomes, in a consecutive series of patients who underwent third‐generation PFA at a non‐designer centre. Additionally, the impact of prior surgery on postoperative clinical outcomes is explored. Based on these considerations, it is hypothesised that PFA performed in this setting achieves satisfactory outcomes, while prior surgical interventions may influence postoperative clinical results.

## METHODS

The study retrospectively analyses prospectively collected data from a consecutive series of patients who underwent PFA for isolated PFOA at a single institution between 2016 and 2023 using a gender‐specific third generation PFA (Gender Solutions® Patello‐Femoral Joint System, Zimmer, Warsaw, IN, USA). Ethical approval was obtained by the institutional review boards of the University of Heidelberg (S‐079/2023) and the study was conducted in accordance with the Helsinki Declaration of 1975, as revised in 2013. Informed consent was obtained from all participating patients.

### Indication, Inclusion and exclusion criteria

Inclusion criteria comprised a minimum age of 18 years and signed informed consent. Patients with a follow‐up (FU) of less than 1 year were excluded.

The indication for surgery was severe OA of the patellofemoral compartment with full thickness articular cartilage loss, either primary or secondary to trauma or patellofemoral instability. Contraindication were the presence of tibiofemoral OA, preoperative range‐of‐motion (ROM) less than 90° and inflammatory disease. Coexistent patellofemoral instability was not considered a contraindication and was treated with additional stabilising procedures if necessary.

In all cases preoperative radiological assessment included radiographs as follows: anteroposterior (AP) and lateral weight bearing, sunrise view, varus/valgus stress (to screen for tibiofemoral OA) and full‐length standing AP. An magnetic resonance imaging was available in some cases, but it was not mandatory for determining the indication.

### Surgical technique and rehabilitation

Surgeries were performed using minimally invasive techniques (MIS) through a medial parapatellar approach. The femoral and patellar component were both cemented. An intravenous single‐shot antibiotic (1.5 g cefuroxime) was administered perioperatively. All procedures were performed by or under supervision of seven senior surgeons with high experiences in arthroplasty.

Postoperative rehabilitation was standardised, allowing immediate full weight bearing and unrestricted knee movement, followed by 3 weeks of rehabilitation.

### Clinical and radiographic assessment

The Oxford Knee Score (OKS) and Forgotten Joint Score (FJS) were obtained at the regular follow‐up (FU) examination. These regular FU examinations are routinely performed at 1, 3 and every 5 years post‐operatively in all patients receiving an arthroplasty at our institution.

Pain level was assessed using a visual analogue scale (VAS) ranging from 0 to 10 (0 = no pain, 10 = worst pain experienced). Postoperative satisfaction was evaluated using a numeric scale ranging from 5 (highly satisfied) to 1 (unsatisfied). The Tegner activity score (TAS) and the University of California Los Angeles activity scale (UCLA) was used to assess patients' physical activity after surgery.

Standardised postoperative radiographs included AP and lateral weight bearing as well as sunrise view. The radiographs were analysed by two examiners (MH and TR) with focus on radiological signs of loosening of the components and progression of OA in the tibiofemoral joint. The OA grade was assessed using the Kellgren and Lawrence classification (KL) [13].

Patients were further grouped based on whether they had prior surgery on the affected knee to compare clinical outcomes.

Patients who were unable to attend the clinical FU were contacted by telephone for a structured interview to assess the aforementioned questionaries.

### Statistical analysis

Data were collected and analysed using SPSS version 29.0 (SPSS Inc., Chicago, IL) and GraphPad Prism version 10.2.2 (GraphPad Software, San Diego, CA). The primary endpoint of the study was implant survival, while secondary endpoints included clinical outcomes. Survivorship analysis was performed using the endpoint 'revision for any reason' and 'reoperation'. Revision surgery was defined as any reoperation in which at least one component was replaced. Continuous variables were reported as mean and standard deviation (SD), with differences between preoperative and postoperative values analyzed using the Wilcoxon Signed Rank test. The percentage of patients who achieved the Patient Acceptable Symptom State (PASS) for the OKS ( > 31.3) and FJS‐12 ( > 40.63) was calculated based on previously published thresholds for unicompartmental knee arthroplasty (UKA), as no specific PASS values for PFA are currently available [11, 39].

Comparisons between patients with prior surgery and those without were conducted using the Mann–Whitney *U* test. Pearson correlation analysis was used to assess potential relationships between BMI, age at the time of surgery, and clinical outcomes.

Survivorship analysis was performed with the Kaplan–Meier estimator. A post hoc power analysis for the Wilcoxon Signed‐Rank test, based on the OKS, revealed a power of 0.999 ( ≈ 100%), indicating a very high probability of detecting a clinically relevant effect at a significance level of 0.05. For all statistical tests, a significance level of *p* < 0.05 was applied.

## RESULTS

A total of 27 PFA were implanted in 23 patients using the Gender Solutions prosthesis between 2016 and 2023. No patient was lost‐to‐follow‐up or deceased during the study period. Seventeen patients (20 PFAs) attended for a clinical FU, while six patients (7 PFAs) were contacted for a structured interview by telephone. The mean FU was 4.3 ± 2.1 years. Patients' demographics are presented in Table 1.

**Table 1:** Clinical and demographic data of the study group.

Demographics	
Mean age at time of surgery in years ± SD	51.7 ± 10.5 (range: 35–69)
Sex (%)	Female: 21 (92.6%); male: 2 (7.4%)
Operated side (%)	Left 16 (59.3%); right 11 (40.7%)
Mean body mass index (kg/m^2^) ± SD	28.3 ± 4.5 (range: 21.2–43.7)

Abbreviation: SD, standard deviation.

### Survivorship analysis

**Figure 1:**
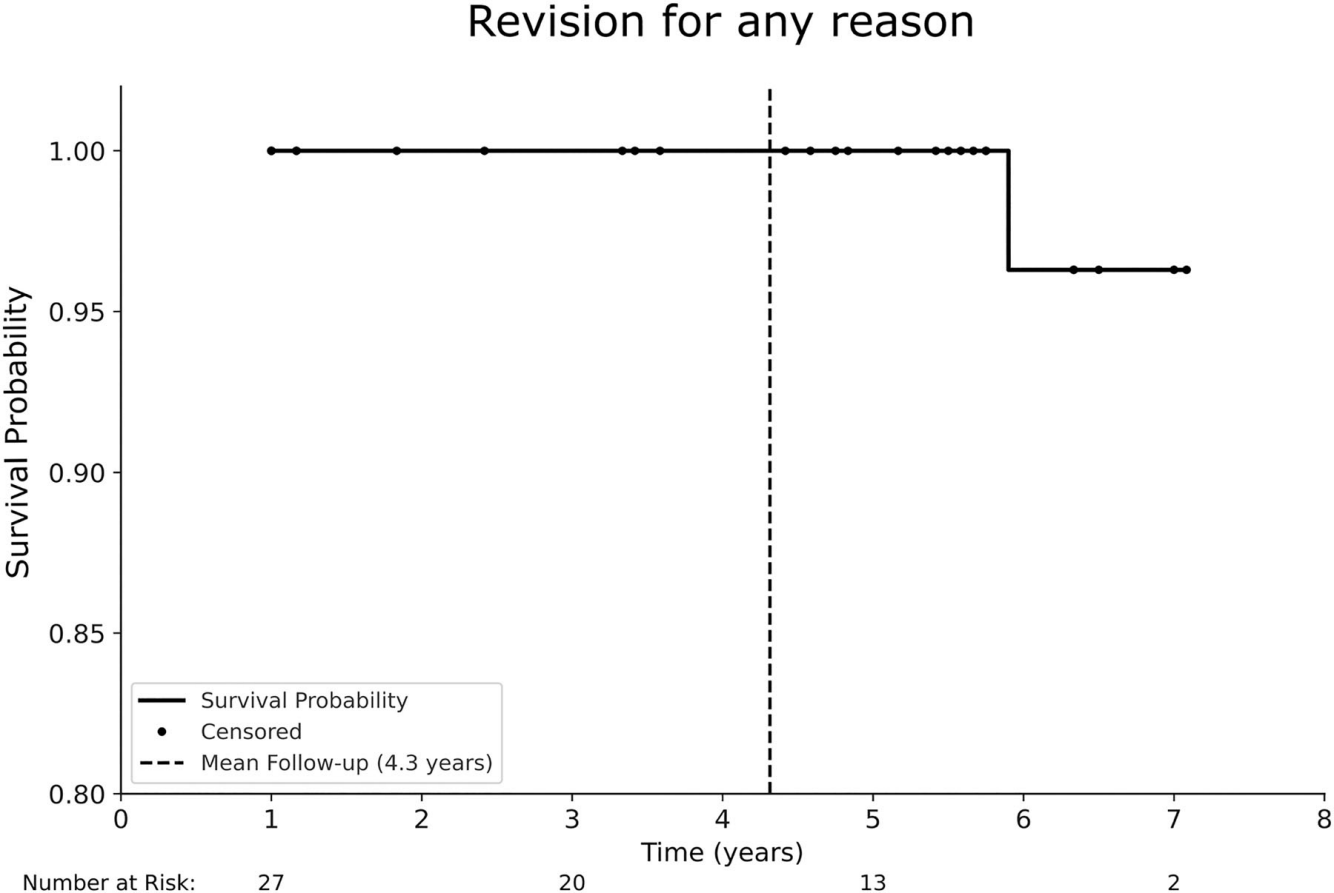
Kaplan–Meier survivorship curve for 'revision for any reason' as the endpoint. The 5‐year survival rate was 100% (95% CI: 87.5–100) with a mean follow‐up time of 4.3 years. CI, confidence interval.

The 5‐year survival rate for the endpoint 'revision for any reason' was 100% (95% confidence interval [CI]: 87.5–100; number at risk: 13) (Figure 1). One revision surgery occurred 6 years post‐operatively due to progression of tibiofemoral OA, necessitating conversion to TKA.

**Figure 2:**
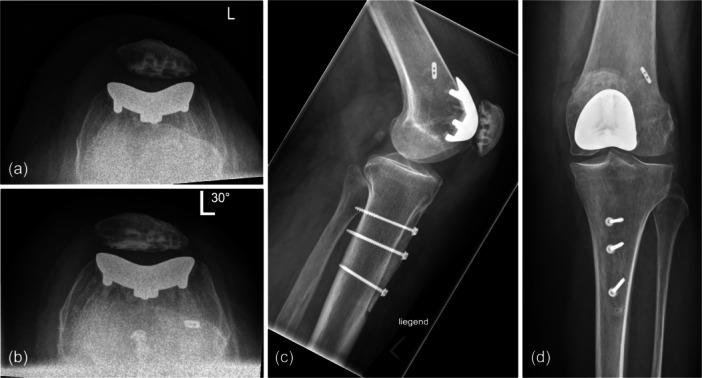
Radiographs of a patient who underwent reoperation for recurrent patellar luxation in full extension 4 months post‐operatively. The treatment included medial patellofemoral ligament (MPFL) augmentation combined with a medializing tibial tubercle osteotomy (TTO). (a) Sunrise view in 30° knee flexion approximately 3 months post‐operatively, showing lateral patellar tilt. (b) Sunrise view after reoperation, demonstrating centralised patellar tracking in 30° knee flexion. (c, d) Lateral and anteroposterior views following reoperation, clearly illustrating the medializing TTO.

The same patient also underwent PFA on the contralateral knee, where she experienced recurrent patellar luxation in full extension shortly after surgery. This required reoperation four months post‐operatively, involving medial patellofemoral ligament (MPFL) augmentation and a medializing tibial tubercle osteotomy (TTO) (Figure 2). No further surgeries were necessary up to the most recent follow‐up, and the patient demonstrated a favourable OKS of 37 five years after the non‐revised PFA. This patient had no history of prior surgery on either knee before undergoing PFA.

**Figure 3:**
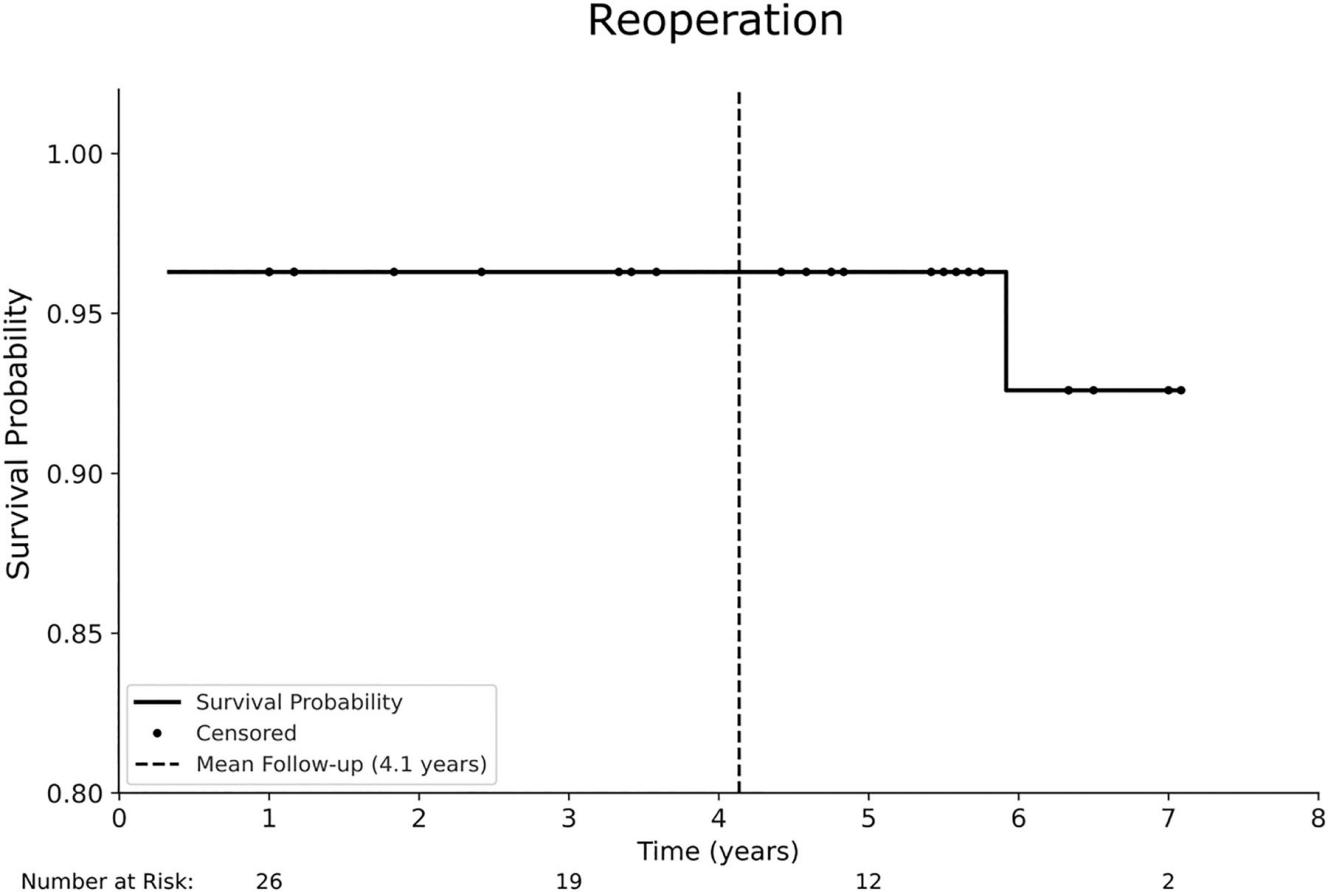
Kaplan–Meier survivorship curve for 'reoperation' as the endpoint. The 5‐year survival rate was 96.3% (95% CI: 76.5–99.5) with a mean follow‐up time of 4.1 years. CI, confidence interval.

The 5‐year survival rate for the endpoint 'reoperation' was 96.3% (95% CI: 76.5–99.5; number at risk: 12) (Figure 3).

### Clinical outcome

**Figure 4:**
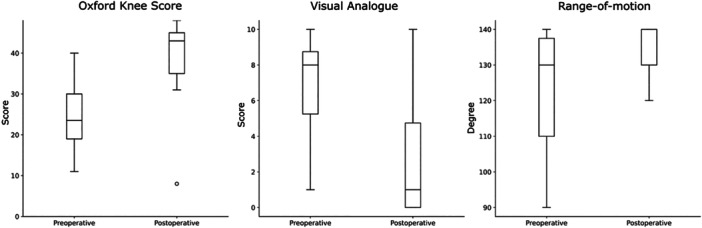
Oxford Knee Score, Visual Analogoue Scale and range‐of‐motion preoperatively and at minimum 12 months follow‐up. The differences were statistically significant (*p* < 0.05).

All non‐revised patients (*n* = 26) were included in this analysis. Postoperative ROM data were available only for patients who attended clinical FU (*n* = 19). Statistically significant improvements were observed in OKS (from 24.7 ± 8.0 to 39.2 ± 8.3, *p* < 0.001), VAS (from 7.1 ± 2.3 to 2.3 ± 2.8, *p* < 0.001) and ROM (from 122.1 ± 17° to 134.7 ± 6.8°, *p* = 0.007) (Figure 4).

Based on OKS criteria, 53.8% of patients achieved an excellent outcome (score > 41), 30.8% had a good outcome (score 34–41), 11.5% had a fair outcome (score 27–33), and 3.8% had a poor outcome (score <27) at the final FU. All assessed parameters are presented in Table 2. 24 of 26 cases (92.3%) attained the PASS for the OKS, while 18 of 26 cases (69.2%) attained the PASS for the FJS‐12.

**Table 2:** Clinical results of the study group.

	Preoperative	Postoperative	Included cases
	Mean ± SD (range)	Mean ± SD (range)	
OKS*	24.7 ± 8.0 (11–40)	39.2 ± 8.3 (8–48)	26
ROM*	122.1 ± 17 (90–140)	134.7 ± 6.8 (120–140)	19
VAS*	7.1 ± 2.3 (1–10)	2.3 ± 2.8 (0–10)	26
FJS	‐	60.8 ± 35.3 (10–100)	26
TAS	‐	2.8 ± 1.4 (0–5)	26
UCLA	‐	5.2 ± 1.8 (1–7)	26

Abbreviations: FJS, forgotten joint score; OKS, oxford knee score; ROM, range‐of‐motion; SD, standard deviation; TAS, tegner activity scale; UCLA, University of California Los Angeles activity scale; VAS, visual analogue scale.

* *p* < 0.05.

Overall, 92.3% of patients reported being satisfied or highly satisfied with the outcome, 3.9% were fairly satisfied, and 3.9% were unsatisfied. A negative correlation was observed between BMI and postoperative ROM (Pearson correlation coefficient: −0.053, *p* = 0.016), as well as between age and postoperative satisfaction rate (Pearson correlation coefficient: 0.400, *p* = 0.039). No other metrics showed significant correlations with BMI or age at the time of surgery.

### Radiological analysis

Preoperatively, the KL grade in the patellofemoral compartment was Grade 4 in 56% (*n* = 15) of cases, Grade 3 in 33% (*n* = 9) and Grade 2 in 11% (*n* = 3). Postoperative radiographic analysis was conducted for all non‐revised patients who attended the outpatient clinic for FU (19 PFAs). One female patient exhibited symptomatic OA progression, primarily in the lateral tibiofemoral compartment, seven years post‐operatively. Although conversion to TKA was recommended, the patient declined revision at the most recent FU. Among the other non‐revised patients, no signs of OA progression or implant loosening were observed. Thus, there were two cases of tibiofemoral OA progression (7.4%), with one patient (3.7%) undergoing revision during the study period

In ten cases (37.0%), at least one prior surgical procedure had been performed on the affected knee joint. The specific procedures performed are detailed in Table 3.

**Table 3:** Surgical history of patients prior to PFA.

Case	Underlying diagnosis	Procedures	Preoperative OKS	Postoperative OKS
#1	Patellofemoral instability	Medial capsular plication	21	32
#2	Patellofemoral instability	Microfracture surgery on trochlea groove, lateral release, medial capsular plication	26	37
#3	Trauma‐induced patellar dislocation	Medial patellofemoral ligament (MPFL) reconstruction	17	35
#4	Patellofemoral instability	Medializing and distalizing tibial tubercle osteotomy (TTO)	11	37
#5	Patellofemoral instability	MPFL‐Reconstruction, retropatellar microfracture surgery	19	44
#6	Patellofemoral instability	MPFL‐Reconstruction, Medializing and distalizing TTO, retropatellar microfracture surgery, lateral release, patellar denervation	35	45
#7	Osteochondrosis dissecans of the retropatellar surface	Osteochondral fixation with screws	34	45
#8	Patellofemoral instabilty	Lateral release, retropatellar microfracture surgery	12	34
#9	Degenerative medial meniscus lesion with retropatellar cartilage damage	Medial meniscus partial resection, retropatellar chrondroplasty	15	48
#10	ACL Rupture with retropatellar cartilage damage	ACL Reconstruction, retropatellar chrondroplasty	22	44

Abbreviations: ACL, anterior cruciate ligament; MPFL, medial patellofemoral ligament; OKS, oxford knee Score; TTO, tibial tubercle osteotomy.

Patients were divided into two groups based on prior surgical history: Group 1 included patients who had undergone prior surgery on the affected knee, while Group 2 consisted of those without previous surgery. Patients in Group 1 were significantly younger than those in Group 2 and reported a higher postoperative satisfaction rate. Additionally, Group 1 demonstrated a significantly greater improvement in ROM (Table 4).

**Table 4:** Comparative demographics and outcomes between Group 1 (prior surgery on the affected knee) and Group 2 (no prior surgery) based on grouping by prior surgery on the affected knee.

	Group 1 (*n* = 10)	Group 2 (*n* = 17)	*p* value
	(Mean ± SD)	(Mean ± SD)	
Age at surgery*	44.6 ± 7.7	55.8 ± 9.7	0.005
BMI	29.8 ± 6.3	27.4 ± 2.9	0.235
Follow‐up in years	3.4 ± 2.2	4.8 ± 1.8	0.108
ROM preoperative	109.3 ± 18.8	125.0 ± 14.3	0.079
ROM postoperative	134.3 ± 7.9	134.6 ± 6.6	1.000
ΔROM*	25.0 ± 16.1	8.1 ± 15.8	0.041
OKS preoperative	21.2 ± 8.3	26.6 ± 7.0	0.066
OKS postoperative	40.1 ± 5.6	38.4 ± 9.3	0.899
ΔOKS	18.9 ± 8.0	11.8 ± 10.9	0.125
VAS preoperative*	8.7 ± 0.8	6.2 ± 2.3	0.005
VAS postoperative	2.6 ± 2.7	2.0 ± 2.7	0.566
ΔVAS	−6.1 ± 3.1	−4.3 ± 3.5	0.180
FJS postoperative	68.4 ± 38.6	56.1 ± 33.5	0.255
TAS postoperative	3.0 ± 1.5	2.7 ± 1.3	1.000
UCLA postoperative	4.9 ± 1.9	5.4 ± 1.8	0.308
Satisfaction postoperative*	4.3 ± 0.8	3.4 ± 1.0	0.035

Abbreviations: BMI, body mass index; FJS, forgotten joint score; OKS, oxford knee score; ROM, range‐of‐motion; SD, standard deviation; TAS, tegner activity scale; UCLA, University of California Los Angeles activity scale; VAS, visual analogue scale.

## DISCUSSION

The main findings of this study indicate a high survival rate of 100% with the endpoint 'revision for any reason' for PFA and good to excellent clinical outcomes in over 85% of patients at short‐ to mid‐term follow‐up with a gender‐specific implant in a non‐designer series.

This implant survivorship is higher than previously published case series on various second‐generation PFAs, which report survival rates between 77% and 97% [2, 3, 19, 22].

The reduction in revision rates for second‐generation PFAs compared to the poor outcomes of first‐generation designs (with revision rates up to 50%) is largely attributed to implant modifications addressing maltracking and trochlear dysplasia [36]. These second‐generation PFAs are characterised by features such as a wider trochlear groove, a valgus tracking angle, and congruent motion throughout the full range of motion without impingement on the anterior cruciate ligament [1, 38]. Although there are design‐specific similarities, registry data indicate that revision rates for second‐generation PFAs vary, with failure probabilities ranging from 6.98% to 12.85% at five years of follow‐up [28]. In the National Joint Registry (NJR) for England, Wales, Northern Ireland, and the Isle of Man, the Gender Solutions prosthesis was the most commonly used PFA, accounting for approximately 50% of cases and demonstrating the lowest cumulative revision rate at 6.4% over 5 years [26]. While registry data provide valuable insights through large datasets, they do not analyse specific factors contributing to positive outcomes, especially for rare indications such as PFA. Cohort studies are thus essential for identifying these factors.

Romagnoli and Marullo reported the largest case series using the Gender Solutions prosthesis from a designer‐related centre, involving 105 PFAs, 41 of which were combined with medial UKA and PFA [27]. The overall survival rate in their study, 95.2% at a mean follow‐up of 5.5 years, was comparable to the present study [27]. Only two other independent studies reported on the short‐term survivorship of this same prosthesis, with survival rates of 95.6% in 52 cases and 96% in 57 cases, respectively [12, 21]. Collectively, registry and cohort study data demonstrate the lowest revision rates for this implant design at short‐term follow‐up.

As extensively reported, progression of OA in the tibiofemoral joint is the most common reason for revision following PFA, as observed in two patients in the present study, one of whom required conversion to TKA [6, 29]. OA progression is not necessarily attributable to surgical or technical errors and may instead be a natural consequence of aging [28].

However, thorough preoperative assessment of the tibiofemoral joint is essential when considering PFA to minimise the risk of early revision. Notably, the use of PFA in younger patients can delay the need for TKA along with its associated risks, and subsequent revision to TKA does not compromise TKA outcomes [17, 23].

In the present study, one patient experienced patellar luxation in full extension and required additional patellar stabilisation with MPFL augmentation and a medializing TTO. Although modifications in second‐generation PFA have significantly reduced patellar maltracking, it remains a concern in contemporary PFA designs. Thienpont et al. also reported patellar alignment issues in a series of 57 patients who underwent PFA with the Gender Solutions prosthesis; two patients exhibited lateral patellar luxation, while four patients had a lateral shift of the patella [33]. Although none of these cases required revision surgery, one patient underwent a realignment TTO with a lateral release [33]. Thienpont et al. analyzed the impact of anatomic variability in the distal trochlea on the coronal alignment of the trochlear component in PFA, noting that condylar anatomy is commonly used to position the femoral component. They found that this approach occasionally resulted in proximal varus alignment of the trochlear component, which correlated with an increased incidence of patellar maltracking [33]. After adjusting the surgical technique to position the trochlear implant according to the AP axis, independent of condylar anatomy, they observed no further cases of patellar maltracking [33].

In terms of clinical outcomes, approximately 85% of patients achieved good to excellent OKS results post‐operatively, and around 92% reported satisfaction with the outcome. The mean postoperative OKS is slightly higher than previously reported outcomes for various second‐generation PFAs, although the difference is not expected to be clinically significant [14, 19, 25]. The postoperative activity level can be considered average and aligns with previous findings on PFA outcomes [7, 9, 18].

A further challenging aspect in knee arthroplasty is the impact of prior surgeries, as these can lead to compromised soft tissue and an increased risk of adverse outcomes, including greater pain, stiffness and infection [24]. Patients with severe PFOA and underlying patellofemoral instability often have a history of previous stabilising procedures. Kazarian et al. found in a study of 57 PFAs (Gender Solutions prosthesis) that prior surgery was associated with worse clinical outcomes [12]. While these findings may seem intuitive, the current study demonstrated the opposite: patients with prior surgery on the affected knee reported significantly higher satisfaction rates and greater postoperative improvement in ROM. Functional outcomes, as measured by the OKS, were also higher in this group, though the difference did not reach statistical significance. The younger age of this group may partially explain their higher functional scores, though younger patients also tend to have higher expectations for activity and functional outcomes [12]. Although further studies with larger sample sizes are needed to clarify the effect of prior surgery on PFA outcomes, the data from the present study suggest that these patients can achieve promising results and therefore should not be excluded as candidates for PFA.

Previous studies have shown that obesity is associated with higher revision rates in patients undergoing PFA, primarily due to OA progression [18]. However, the impact of obesity on functional outcomes remains unclear, as earlier studies have reported contrasting findings [9, 35]. In the present study, BMI was associated with a slight negative effect on postoperative ROM, though this effect was not considered clinically relevant. Thus, we suggest that obesity should not be regarded as an absolute contraindication for PFA.

The primary limitations of this study include its retrospective design and the relatively small sample size. Especially when analysing the impact of prior surgery on postoperative outcomes, the sample size may be underpowered.

One limitation of this study is the varying follow‐up durations, which resulted in censoring of some patients in the survival analysis. However, the Kaplan–Meier method appropriately accounts for censoring, ensuring that these cases do not artificially lower the estimated survival probabilities. Additionally, the minimum follow‐up of 1 year likely provided sufficient time for patient rehabilitation. Furthermore, patient‐reported outcome measures were collected at varying time points, ranging from 1 to 7 years post‐operatively. Consistent data collection at the same time point for all patients would have strengthened the findings. Finally, selection bias may be present, as approximately one‐quarter of patients did not attend our outpatient clinic and were assessed only via telephone.

## CONCLUSION

The short‐ to mid‐term results following third‐generation PFA demonstrated high survivorship and excellent clinical outcomes in an independent series. Prior surgery and obesity were not associated with poorer clinical outcomes, supporting the consideration of PFA for these patients when appropriately indicated.

## AUTHOR CONTRIBUTIONS

All authors contributed to the study conception and design. Mustafa Hariri contributed to acquisition, analysis and interpretation of data and drafted the manuscript and figures. Hannah Schwab, Paul Mick, Johannes Weishorn and Kevin‐Arno Koch performed data collection and revised the manuscript critically. Timo Nees and Tobias Reiner performed the statistical analysis and revised the manuscript critically. Timo Nees and Tilman Walker participated in the study design and helped to draft the manuscript. TR drafted the manuscript and revised it critically.

## CONFLICT OF INTEREST STATEMENT

The authors declare no conflicts of interest.

## ETHICS STATEMENT

Ethical approval was obtained by the institutional review boards of the University of Heidelberg (S‐079/2023) and the study was conducted in accordance with the Helsinki Declaration of 1975, as revised in 2013. Informed consent was obtained from all participants included in the study.

## Data Availability

The data will be available upon reasonable request.
